# Therapeutic potential of casticin in alleviating ovarian torsion/detorsion-induced ovarian and lung injuries

**DOI:** 10.22038/ijbms.2025.85990.18572

**Published:** 2025

**Authors:** Ersen Eraslan, Ayhan Tanyeli̇, Mustafa Can Güler, Fazile Nur Eki̇nci̇ Akdemi̇r, Ömer Topdaği, Şahin Yazici, Derya Güzel Erdoğan, Esra Çinar Tanriverdi̇, Selim Çomakli

**Affiliations:** 1 Department of Physiology, Faculty of Medicine, Bandırma Onyedi Eylül University, Bandırma, Turkey; 2 Department of Physiology, Faculty of Medicine, Atatürk University, Erzurum, Turkey; 3 Department of Nutrition and Dietetics, Faculty of Health Science, Ağrı İbrahim Çeçen University, Ağrı, Turkey; 4 Department of Internal Medicine, Faculty of Medicine, Bahçeşehir University, Istanbul, Turkey; 5 Department of Obstetrics and Gynecology, Erzurum City Hospital, Erzurum, Turkey; 6 Department of Physiology, Faculty of Medicine, Sakarya University, Sakarya, Turkey; 7 Department of Medicine Education, Atatürk University, Erzurum, Turkey; 8 Department of Pathology, Faculty of Veterinary Medicine, Atatürk University, Erzurum, Turkey

**Keywords:** Casticin, Torsion detorsion, Ovary, Lung, Rat

## Abstract

**Objective(s):**

Ovarian torsion is a critical gynecological emergency caused by the twisting of the ovary around its supporting structures, resulting in compromised blood flow and potential ovarian damage. Ovarian torsion/detorsion (T/D) can lead to severe complications, including infertility, if not promptly addressed. This study aimed to investigate the therapeutic effects of casticin (CAST) on ovarian and lung tissue injuries induced by a bilateral ovarian T/D model in rats.

**Materials and Methods:**

Experimental animals were randomly allocated into groups of sham, T/D, CAST 5 mg/kg, and CAST 10 mg/kg. Ovarian and lung tissues were subjected to biochemical, histopathological, and immunohistochemical analyses.

**Results:**

In the T/D group, markers of oxidative stress and inflammation, including myeloperoxidase (MPO) activity, malondialdehyde (MDA), oxidative stress index (OSI), tumor necrosis factor-alpha (TNF-α), and 8-hydroxy-2’ deoxyguanosine (8-OHdG), were significantly elevated (*P<*0.05). Conversely, superoxide dismutase (SOD) activity and total antioxidant status (TAS) levels were notably decreased (*P<*0.05). CAST administration significantly attenuated tissue damage by reducing oxidative stress and inflammatory markers while enhancing antioxidant defense (*P<*0.05).

**Conclusion:**

CAST demonstrated a therapeutic effect against ovarian and lung tissue damage induced by T/D, suggesting that it may serve as a potential therapeutic agent for treating ovarian T/D-related injuries. These findings underscore the potential of CAST in clinical settings, particularly as a novel intervention to mitigate complications associated with I/R injuries in gynecological emergencies.

## Introduction

Ovarian torsion (O/T) is a gynecological emergency that affects women of all age groups, with significant implications for fertility, particularly in women of reproductive age. O/T involves the partial or complete rotation of the ovary around its ligamentous structures, compromising blood flow and resulting in ischemic damage (1, 2). Prompt surgical intervention, either through laparotomy or laparoscopy, can restore perfusion via detorsion in O/T (3). However, while detorsion alleviates ischemia, it also introduces ischemia-reperfusion (I/R) injury, primarily mediated by the excessive production of reactive oxygen species (ROS)(4). Ovarian I/R injury may damage remote organs, including the lungs (5).

ROS-induced lipid peroxidation produces malondialdehyde (MDA), a biomarker of oxidative stress and cellular damage. Elevated MDA levels and increased oxidative stress exacerbate tissue injury by triggering a cascade of cellular and molecular events (6). Myeloperoxidase (MPO), an enzyme predominantly found in neutrophils, is pivotal in mediating oxidative stress and tissue damage during inflammation. Elevated MPO activity is strongly associated with generating hypochlorous acid and other reactive species, exacerbating cellular injury and inflammation (7). Additionally, 8-hydroxy-2’ deoxyguanosine (8-OHdG), a marker of DNA oxidative damage, is frequently used to indicate oxidative stress (8).

The inflammatory response following I/R injury is marked by the up-regulation of proinflammatory cytokines, including tumor necrosis factor-alpha (TNF-α), which contributes to neutrophil activation and increased ROS levels (9, 10). Antioxidant defense mechanisms, such as the activity of superoxide dismutase (SOD), play a critical role in mitigating ROS-induced damage (11). 

Casticin (CAST, 3′, 5-dihydroxy-3, 4′, 6, 7-tetramethoxyflavone) is a flavonoid derived from *Vitex Fructus*, a plant widely used in traditional medicine (12). Studies have demonstrated its antioxidant and anti-inflammatory properties (13, 14). CAST suppresses the production of the proinflammatory cytokine tumor TNF-α, highlighting its potential anti-inflammatory properties (15). CAST has been shown to inhibit ROS production and MDA generation in models of ulcerative colitis (16) and reduce MPO activity in acute lung injury by mitigating oxidative stress and neutrophilic inflammation (17). However, its potential role in addressing the oxidative and inflammatory damage in ovarian T/D remains unexplored.

In the present study, we investigated whether CAST could alleviate oxidative and inflammatory damage associated with ovarian T/D, focusing on its potential role in reducing tissue injury in ovarian and lung tissues.

## Materials and Methods

### Chemical materials

Ketamine (Ketalar®, Pfizer, Istanbul) and xylazine hydrochloride (Rompun®, Bayer, Istanbul) were preferred for anesthesia. CAST (purity ≥95.0%, CAS Number: 479-91-4) was purchased from Sigma Aldrich (Germany). A 10% povidone-iodine solution (Batticon; Adeka) was used for disinfection.

### Ethical approval

Atatürk University Experimental Animal Ethics Committee allowed the study (protocol no:28.06.2018/137). The experimental process was carried out following ARRIVE guidelines (https://arriveguidelines.org). The study ensured the ethical use of animals by minimizing harm, optimizing study design, and adhering to replacement, reduction, and refinement principles.

### Experimental animals

The experimental animals were procured from Atatürk University Experimental Animals Research and Application Center (ATADEM). They were housed in cages with appropriate laboratory conditions (balanced temperature ~22 ^°^C, mean humidity ~55%, and controlled 12:12 light-dark). Rats were fed standard rat feed and provided with drinking water. All animals were deprived of food 24 hr before the experiment, but were allowed to drink water.

### Preoperative protocol

**Figure 1:**
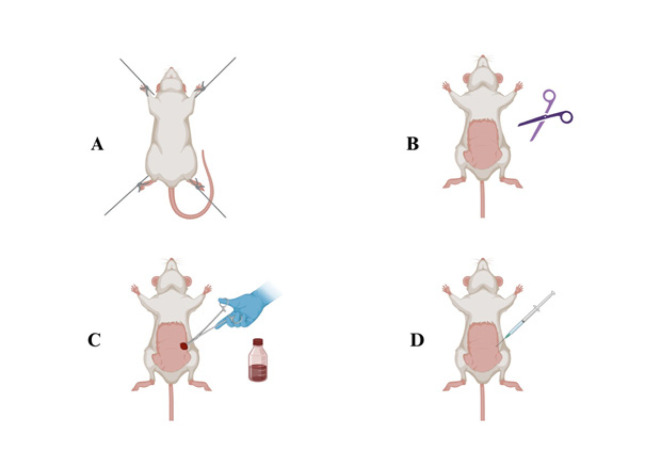
Preoperative preparation of the rats for the ovarian T/D experimental model

The rats were placed in the supine position for stabilization (Figure 1a). The incision region was carefully shaved (Figure 1b). Following disinfection with 10% povidone-iodine solution (Figure 1c), anesthesia was induced using ketamine/xylazine (Figure 1d). Ketamine/xylazine anesthesia doses (100/15 mg/kg body weight (BW), intraperitoneal (IP)) were based on a previously validated protocol (18). No mortality or anesthetic complications were observed in our experimental study.

### Groups and torsion/detorsion model

**Figure 2:**
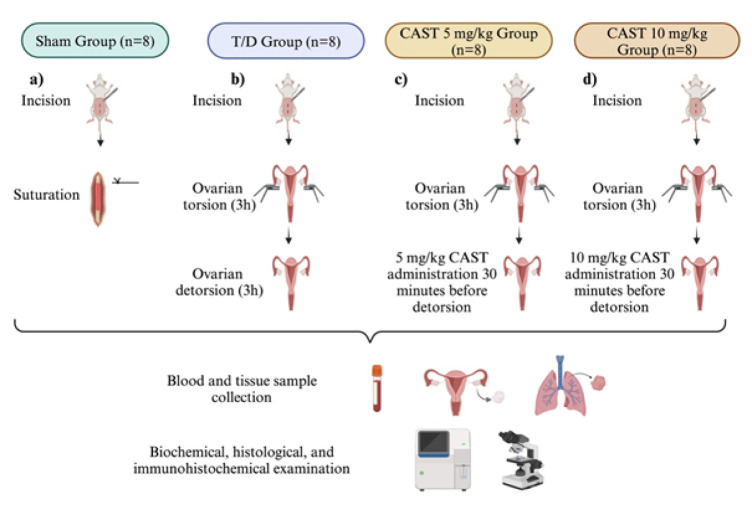
Experimental rat groups and procedures in the ovarian T/D experimental model

All experimental processes were performed in ATADEM. Thirty-two female Sprague-Dawley rats (12-16 weeks old, 200-250 g) were grouped as follows (Figure 2).

Sham Group (n=8): A 1-2 cm midline incision was made to open the abdominal area and sutured immediately with silk 3/0 suture (Figure 2a). 

T/D group (n=8): The same surgical steps were followed as described in the sham group. Before suturation, torsion was established as described in previous research (19). The ovaries, fallopian tubes, and related structures were rotated clockwise 360 degrees and fixed via atraumatic clamps for three hours. Later, the detorsion phase started by releasing clamps to provide blood flow for three hours (Figure 2b). 

CAST 5 mg/kg (n=8): The T/D model was carried out as described in the T/D group. In addition, 5 mg/kg CAST (20) was administered to the rats intraperitoneally (IP) 30 min before detorsion (Figure 2c). 

CAST 10 mg/kg (n=8): 10 mg/kg CAST (20) was given to the rats IP, 30 min before detorsion (Figure 2d). 

Following the experiment, the rats were sacrificed as described (21, 22). Rats were euthanized by deep anesthesia using intravenous sodium thiopental (150 mg/kg BW), followed by isoflurane exposure in a sealed chamber, following AVMA guidelines (2020). The ovarian and lung tissues were excised and stored for analysis. Following excision, tissues for biochemical analysis were immediately frozen in liquid nitrogen and stored at -80 ^°^C until analysis. Tissues designated for histological and immunohistochemical evaluation were fixed in 10% buffered formaldehyde solution at room temperature.

### Biochemical analysis

Tissue samples (100 mg each) were homogenized in phosphate-buffered saline (PBS), and the homogenates were centrifuged to obtain supernatants, which were stored at -80 ^°^C until analysis. SOD, MPO, and MDA levels were measured using commercial kits from Thermo Fisher Scientific (USA).

MDA levels were determined by quantifying the compound formed through the reaction between MDA and thiobarbituric acid, as described previously (23). MPO activity was measured based on the oxidation reaction between MPO and o-dianisidine, which forms a complex indicative of MPO levels (24). SOD activity was assessed by measuring the formation of formazan dye, with activity inversely proportional to dye levels (25). 

Total antioxidant status (TAS) and total oxidant status (TOS) were quantified using commercial kits obtained from Rel Assay Diagnostics (Gaziantep, Turkey). The oxidative stress index (OSI) measures the rate of TOS to TAS and is calculated as OSI=[(TOS, μmol/l)/(TAS, mmol/l)×10](26).

### Histological assessment

Ovarian and lung tissue samples were collected and fixed in 10% formaldehyde solution for 48 hr. Following fixation, the tissues were processed through standard histopathological procedures over 24 hr and embedded in paraffin blocks. The paraffin-embedded tissue samples were sectioned and stained with hematoxylin and eosin (H&E) for microscopic examination.

Histopathological evaluation focused on hemorrhage and degenerative changes in ovarian tissue and the thickness of interalveolar septa in lung tissue. The severity of these changes was scored as none (-), mild (+), moderate (++), or severe (+++)(27). Microscopic examinations were conducted using an Olympus BX51 microscope, and images were captured with DP72 software (Olympus® Inc., Tokyo, Japan) for documentation and analysis.

### Immunohistochemical (IHC) staining

Ovarian and lung tissue samples were dissected and fixed in 10% formaldehyde saline for 24 hr. After fixation, the formaldehyde was removed by washing the tissues thoroughly with tap water. The tissues were then dehydrated in graded alcohol, cleared in two changes of xylene, and embedded in paraffin wax. For immunohistochemical analysis, tissue sections were incubated in 3% hydrogen peroxide (H₂O₂) for 10 min to block endogenous peroxidase activity. Antigen retrieval was performed by heating the sections in an antigen retrieval solution to expose tissue antigens. To minimize nonspecific binding, sections were treated with a blocking solution.

Primary antibodies specific to 8-OHdG (Santa Cruz, Cat. No: sc-66036, Dilution: 1:250) and TNF-α (Novus Biological, Cat. No: NBP1-19532, Dilution: 1:100) were applied to the tissue sections, which were then incubated at room temperature for 60 min. Following incubation, the sections were washed with phosphate-buffered saline (PBS) and treated with a secondary antibody using the Expose Mouse and Rabbit Specific HRP/DAB Detection IHC Kit (Abcam: ab80436). To visualize the immunopositivity, 3,3′-diaminobenzidine (DAB) was used as the chromogen, and hematoxylin was applied as a counterstain. Stained sections were examined under a light microscope, and immunopositivity was graded as none (−), mild (+), moderate (++), or intense (+++) (27).

### Statistical analysis

All statistical analyses were performed using the SPSS 20.0 software package. A *P*-value of <0.05 was considered statistically significant. Biochemical data were analyzed using a one-way analysis of variance (ANOVA), followed by Tukey’s Honest Significant Difference (HSD) test for *post hoc* comparisons among multiple groups. Results are expressed as mean±standard error of the mean (SEM). Histopathological data were assessed using the Kruskal-Wallis test to identify group differences. The Mann-Whitney U test was applied for pairwise group comparisons to determine the specific sources of these differences.

## Results

### Biochemical results

**Figure 3:**
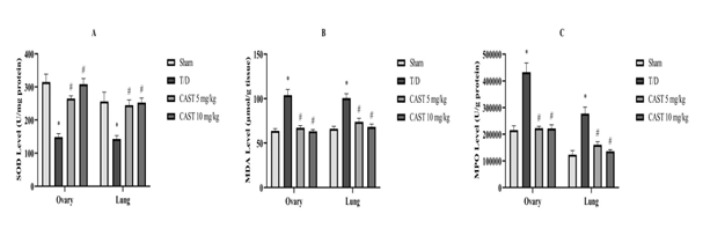
SOD activity (A), MDA levels (B), and MPO activity (C) in ovarian and lung tissues of the rats in the ovarian T/D experimental model

**Figure 4:**
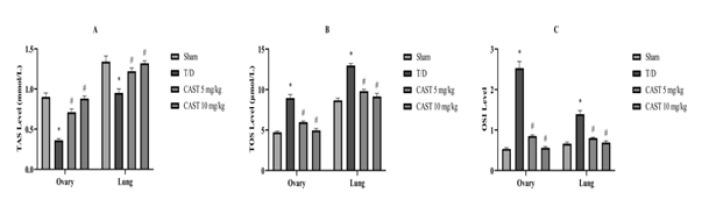
TAS levels (A), TOS levels (B), and OSI (C) in ovarian and lung tissues of the rats in the ovarian T/D experimental model

Biochemical parameters were evaluated in ovarian and lung tissue samples (Figures 3 and 4). The T/D group exhibited a marked reduction in SOD activity and TAS levels in both ovarian and lung tissues compared to the sham group (Figures 3A and 4A, *P*<0.05). Treatment with CAST significantly improved these antioxidant parameters, with no dose-dependent differences observed between the 5 mg/kg and 10 mg/kg doses (Figures 3A and 4A, *P*<0.05).

TOS, OSI, MDA levels, and MPO activity were significantly elevated in the T/D group, indicating enhanced oxidative damage (Figures 3B, 3C, 4B, and 4C, *P*<0.05). Both doses of CAST administration effectively reduced these parameters, but no significant dose-dependent differences were noted (Figures 3B, 3C, 4B, and 4C, *P*<0.05).

### Histological results of ovarian tissue

**Figure 5:**
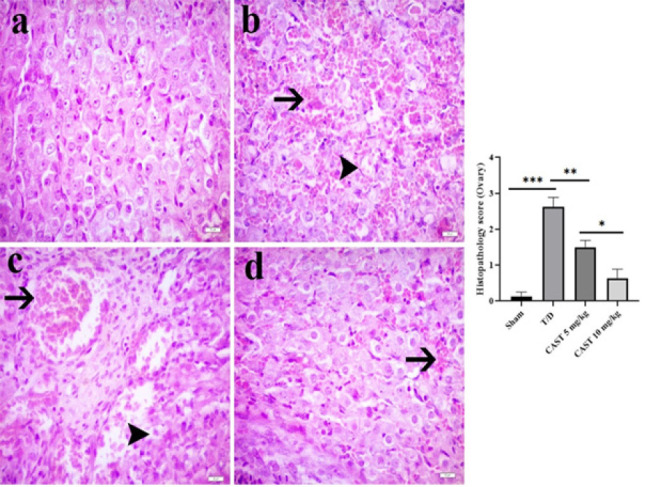
Ovarian tissue samples of the rats in the ovarian T/D experimental model

Histopathological examination revealed no notable changes in lutein cells in the sham group, indicating normal tissue morphology (Figure 5a). In contrast, the T/D group exhibited severe pathological changes, including extensive areas of hemorrhage, significant cellular degeneration, and marked vascular congestion (Figure 5b, *P*<0.05).

There was a noticeable reduction in hemorrhagic areas in the CAST 5 mg/kg group, although degenerative changes in the ovarian tissue were still evident (Figure 5c, *P*<0.05). The CAST 10 mg/kg group showed a more pronounced improvement, with a significant reduction in hemorrhage and minimal degenerative changes observed (Figure 5d, *P*<0.05). Notably, there was a significant difference between the CAST 5 mg/kg and 10 mg/kg groups, with the 10 mg/kg group exhibiting superior outcomes (*P*<0.05).

### Histological results of lung tissue

**Figure 6:**
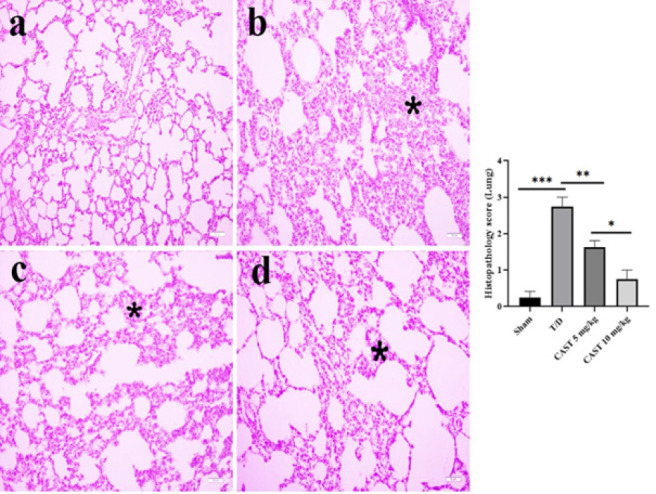
Lung tissue samples of the rats in the ovarian T/D experimental model

Histopathological examination of lung tissue showed that the alveolar walls in the sham group exhibited normal histological architecture with no observable abnormalities (Figure 6a). In the T/D group, severe thickening of the interalveolar septa was observed, primarily due to extensive inflammatory cell infiltration (Figure 6b, *P*<0.05).

In the CAST 5 mg/kg group, the interalveolar thickening was moderately reduced compared to the T/D group, indicating partial amelioration of inflammation (Figure 6c, *P*<0.05). The CAST 10 mg/kg group demonstrated a more significant improvement, with alveolar regions showing essentially normal histological structures and a marked reduction in interalveolar thickening (Figure 6d, *P*<0.05). Notably, there was a significant difference between the CAST 5 mg/kg and 10 mg/kg groups, with the 10 mg/kg group showing superior histological recovery (*P*<0.05).

### Immunohistochemical results of ovarian tissue

**Figure 7:**
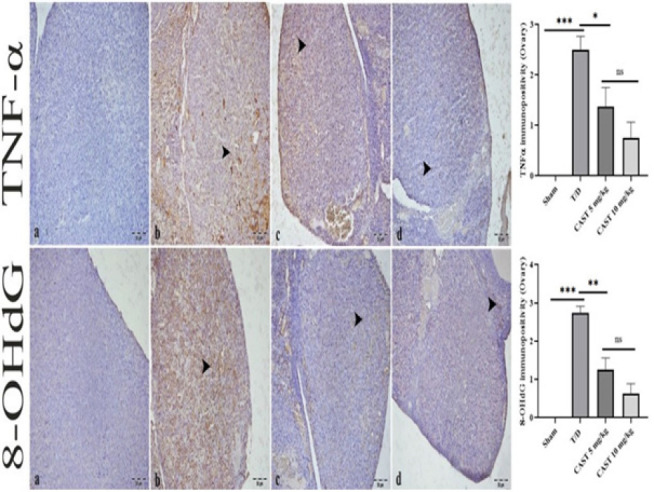
Ovarian tissue samples of the rats in the ovarian T/D experimental model

TNF-α immunopositivity was absent in the sham group, indicating no detectable expression (Figure 7a). In contrast, the T/D group exhibited the most intense TNF-α immunopositivity, a statistically significant increase compared to the sham group (Figure 7b, *P*<0.05). In the CAST groups, TNF-α immunopositivity was significantly reduced relative to the T/D group, with the 5 mg/kg and 10 mg/kg doses showing notable improvement (Figures 7c and 7d, *P*<0.05).

No 8-OHdG immunopositivity was observed in the sham group (Figure 7a). However, intense 8-OHdG immunopositivity was detected in the luteal cells of the T/D group, significantly higher than that of the sham group (Figure 7b, *P*<0.05). In the CAST 5 mg/kg group, a marked reduction in 8-OHdG immunopositivity was observed compared to the T/D group (Figure 7c, *P*<0.05). Furthermore, the CAST 10 mg/kg group exhibited only mild 8-OHdG immunopositivity in the interstitial areas, indicating a substantial improvement relative to the T/D group (Figure 7d, *P*<0.05).

Significant differences existed between the CAST 5 mg/kg and 10 mg/kg groups, with the 10 mg/kg group showing superior histological recovery for reduction in TNF-α and 8-OHdG immunopositivity (*P*<0.05).

### Immunohistochemical results of lung tissue

**Figure 8:**
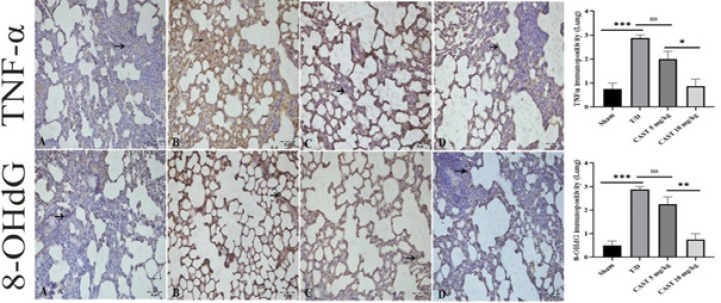
Lung tissue samples of the rats in the ovarian T/D experimental model

Mild TNF-α immunopositivity was observed in the sham group, indicating baseline expression (Figure 8A). The most intense TNF-α immunopositivity was detected in the T/D group and the CAST 5 mg/kg group, showing a significant increase compared to the sham group (Figures 8B and 8C). In contrast, the CAST 10 mg/kg group demonstrated significantly milder TNF-α immunopositivity than the T/D group, indicating a reduced inflammatory response (Figure 8D, *P*<0.05).

Similarly, mild 8-OHdG immunopositivity was observed in the interalveolar areas of the sham group, reflecting minimal oxidative DNA damage (Figure 8A, *P*<0.05). In the T/D group and the CAST 5 mg/kg group, 8-OHdG immunopositivity was intense within the alveolar regions, indicating heightened oxidative stress (Figures 8B and 8C, *P*<0.05). However, in the CAST 10 mg/kg group, 8-OHdG immunopositivity was significantly reduced, with only mild immunopositivity observed in the interstitial areas compared to the T/D group, highlighting the therapeutic effect of CAST (Figure 8D, *P*<0.05).

A significant difference was observed between the CAST 5 mg/kg and 10 mg/kg groups, with the 10 mg/kg group exhibiting more remarkable histological improvement, including a more pronounced reduction in TNF-α and 8-OHdG immunopositivity (*P*<0.05).

## Discussion

Our study demonstrates that ovarian T/D significantly elevates oxidative stress markers, including MDA, MPO, TOS, and OSI while reducing antioxidant defense mechanisms, as evidenced by decreased TAS and SOD levels. Concurrently, TNF-α, an inflammatory mediator, was markedly increased, alongside heightened oxidative DNA damage, indicated by elevated 8-OHdG levels. These results align with previous research emphasizing the contribution of ovarian I/R injury to oxidative stress, inflammation, and the release of proinflammatory cytokines (28, 29). Notably, treatment with CAST effectively reversed these pathological changes by restoring the oxidant-antioxidant balance, reducing inflammation, and mitigating DNA damage. Furthermore, high-dose CAST administration demonstrated superior efficacy to low-dose in alleviating inflammatory responses and oxidative DNA damage. Collectively, these findings highlight CAST’s potential as a promising therapeutic agent for mitigating tissue damage associated with ovarian T/D.

O/T is a prevalent gynecological emergency that poses significant risks of infertility and chronic complications if not treated promptly (30). Surgical detorsion is the primary intervention to restore blood flow and reperfusion to the ovarian tissue (31). However, while reperfusion alleviates ischemia, it paradoxically exacerbates tissue damage through various mechanisms, including the generation of ROS (32). I/R injury is characterized by elevated ROS levels, reduced antioxidant enzyme activity, and oxidative damage, further exacerbating tissue injury (33). 

I/R injury promotes the generation of MDA, a marker of lipid peroxidation and oxidative stress (34), and 8-OHdG expression, a biomarker of oxidative DNA damage (35). In previous studies, MPO activity was significantly elevated in ovarian tissue following I/R, contributing to tissue damage (36). MPO indirectly indicates neutrophil infiltration, further exacerbating oxidative stress and triggering the release of proinflammatory cytokines, including TNF-α (37). In our study, the T/D group caused an increase in oxidative stress markers (MDA, MPO, TOS, and OSI), inflammatory parameters (TNF-α), and oxidative DNA damage (8-OHdG). These findings were similar to the literature.

The antioxidant defense system is critical in neutralizing ROS and maintaining tissue integrity (38). SOD exhibits a high antioxidant capacity and directly regulates cellular ROS levels (39). I/R injury significantly impairs the SOD activity, resulting in a marked decline in its levels within affected tissues. This reduction in SOD activity compromises the tissue’s ability to neutralize ROS, thereby exacerbating oxidative stress and contributing to further cellular and molecular damage (40). In our study, anti-inflammatory parameters (TAS, SOD) declined.

CAST, a natural flavonoid with potent antioxidant and anti-inflammatory properties (41, 42), demonstrated remarkable therapeutic effects against oxidative stress and inflammatory damage induced by T/D in our study. CAST effectively mitigated the increase in oxidative stress markers, including MDA, MPO, TOS, and OSI, which are known to exacerbate cellular and molecular injury following I/R. The reduction in MPO activity observed in CAST-treated groups further underscores its role in limiting neutrophil infiltration and the associated oxidative stress, which are central to the pathophysiology of I/R injury. The ability of CAST to restore TAS and SOD levels indicates its potent antioxidant action, which helps reestablish the oxidant-antioxidant balance, a critical factor in preventing further tissue damage. CAST administration reduced MDA levels and increased SOD activity in ovariectomized rats in literature (43), similar to our data. 

Additionally, CAST significantly reduced the inflammatory mediator TNF-α, the hallmark of I/R-induced inflammatory responses. Consistent with our results, CAST attenuated inflammation by inhibiting the proinflammatory cytokine TNF-α in a chronic obstructive pulmonary disease rat model (44).  CAST alleviated the cascade of inflammation that contributes to tissue injury and organ dysfunction by suppressing the release of proinflammatory cytokines. This anti-inflammatory action underscores its therapeutic potential in mitigating I/R-induced injuries by addressing the key pathways driving inflammation.

Moreover, CAST demonstrated a pronounced capacity against oxidative DNA damage, as evidenced by decreased levels of 8-OHdG. This finding highlights the potential of CAST to preserve the structural integrity and functional capacity of cellular DNA in the face of oxidative stress, similar to the literature (45). Interestingly, the therapeutic effects of CAST were dose-dependent, with higher doses showing greater efficacy in reducing oxidative stress and inflammatory markers and preventing DNA damage.

a) Immobilization of the experimental animal in the supine position, b) Shaving the abdominal region, c) Disinfection of the incision area, d) Anesthesia administration before the experimental model (created with BioRender.com)

a) Sham group, midline incision, and immediate suturing, b) T/D group, torsion for three hours followed by three hours of detorsion, c) CAST 5 mg/kg group, 5 mg/kg CAST administered 30 min before detorsion, d) CAST 10 mg/kg, 10 mg/kg CAST administered 30 min before detorsion (T/D= torsion detorsion, CAST= Casticin), (Created with BioRender.com)

Data are presented as mean±standard deviation. *P<0.05 compared to the sham group; #*P<*0.05 compared to the T/D group. No significant dose-dependent differences were observed between 5 mg/kg and 10 mg/kg CAST groups

SOD= Superoxide dismutase, MDA= malondialdehyde, MPO= myeloperoxidase, CAST= Casticin

Data are presented as mean±standard deviation. *P<0.05 compared to the sham group; #*P<*0.05 compared to the T/D group. No significant dose-dependent differences were observed between 5 mg/kg and 10 mg/kg CAST-treated groups

TAS= Total antioxidant status, TOS= Total oxidant status, OSI= Oxidative stress index, CAST= Casticin

a) Sham, lutein cells with normal histology, b) T/D, severe bleeding (arrow) and degeneration (arrowhead), c) CAST 5 mg/kg, bleeding (arrow) and hydropic degeneration (arrowhead), d) CAST 10 mg/kg, areas of mild bleeding (arrow), 100x H&E (* *P<*0.05, CAST 10 mg/kg group compared to the CAST 5 mg/kg group, ** *P<*0.05, CAST groups compared to the T/D group, *** *P<*0.05, T/D group compared to the sham group

CAST= Casticin, T/D= Torsion/detorsion

a) Sham, lung alveolar structure with normal histology, b) T/D, severe thickening in the interalveolar area (asterix), c) CAST 5 mg/kg, thickening in the interalveolar area (asterix) d) CAST 10 mg/kg, mild thickening in the interalveolar area (asterix) and alveoli with normal histology, 100x H&E (* *P<*0.05, CAST 10 mg/kg group compared to the CAST 5 mg/kg group, **P<0.05, CAST groups compared to the T/D group, ***P<0.05, T/D group compared to the sham group

CAST= Casticin, T/D= Torsion/detorsion

a) Sham group, b) T/D group, intense TNF-α and 8-OHdG immunopositivity in luteal cells-arrowhead, c) CAST 5 mg/kg group, mild TNF-α and 8-OHdG immunopositivitya in luteal cells-arrowhead, d) CAST 10 mg/kg group, mild TNF-α and 8-OHdG immunopositivity in the interstitial space-ok, IHC (* *P<*0.05, CAST 10 mg/kg group compared to the CAST 5 mg/kg group, ** *P<*0.05, CAST groups compared to the T/D group, *** *P<*0.05, T/D group compared to the sham group

CAST= Casticin, T/D= Torsion/detorsion

a) Sham group, mild 8-OHdG immunopositivity in interalveolar area-arrow, b) T/D group, intense 8-OHdG immunopositivity in the alveolar wall-arrow, c) CAST 5 mg/kg group, intense 8-OHdG immunopositivity in the alveolar wall-arrow, d) CAST 10 mg/kg group, mild 8-OHdG immunopositivity in interalveolar area-arrow, IHC. a) Sham group, mild TNF-α immunopositivity in interstitial space-arrow, b) T/D group, intense TNF-α immunopositivity in interstitial space-arrow, c) CAST 5 mg/kg group, intense TNF-α immunopositivity in interstitial space-arrow, d) CAST 10 mg/kg group, mild TNF-α immunopositivity in the alveolar wall-arrow, IHC (* *P<*0.05, CAST 10 mg/kg group compared to the CAST 5 mg/kg group, ** *P<*0.05, CAST groups compared to the T/D group, *** *P<*0.05, T/D group compared to the sham group

CAST= Casticin, T/D= Torsion/detorsion

## Conclusion

CAST demonstrated significant therapeutic efficacy against ovarian T/D injury in this rat model. CAST treatment effectively reduced oxidative stress markers such as MDA and MPO, and improved antioxidant levels including SOD and TAS. Inflammatory markers, including TNF-α, were markedly reduced following CAST administration. CAST alleviated histopathological damage in both ovarian and lung tissues, confirming its systemic therapeutic impact. These results support the clinical potential of CAST as a therapeutic agent for managing complications of ovarian T/D.

The multifunctional properties of CAST make it a compelling candidate for therapeutic use in mitigating the effects of ovarian T/D. Its ability to modulate both oxidative and inflammatory pathways simultaneously provides a comprehensive approach to addressing the complex pathophysiology of ovarian I/R injury. These findings validate the therapeutic potential of CAST and pave the way for future research exploring its use in clinical settings to reduce the long-term complications associated with ovarian T/D. 

## Declaration

We acknowledge the use of Quillbot AI to improve the language and readability of the manuscript.

## Limitations

Despite its promising results, this study has several limitations. First, using a rat model restricts the direct extrapolation of findings to humans due to potential differences in physiology, metabolism, and immune responses. Additionally, the experimental conditions, including controlled dosing and timing, may not entirely reflect clinical scenarios. Another limitation is the lack of long-term outcome data, as the study only evaluated acute responses to CAST treatment. Further research is warranted to address these limitations and advance the translational potential of CAST. Finally, while CAST demonstrated significant efficacy, the absence of comparative analysis with other known treatments limits our understanding of its relative effectiveness.
